# Development, Characterization and In Vitro Biological Properties of Scaffolds Fabricated From Calcium Phosphate Nanoparticles

**DOI:** 10.3390/ijms20071790

**Published:** 2019-04-11

**Authors:** Lizette Morejón, José Angel Delgado, Alexandre Antunes Ribeiro, Marize Varella de Oliveira, Eduardo Mendizábal, Ibrahim García, Adrián Alfonso, Patrina Poh, Martijn van Griensven, Elizabeth R. Balmayor

**Affiliations:** 1 Center of Biomaterials, University of Havana, Havan 10400, Cuba; jadelgado@biomat.uh.cu (J.A.D.); ycachon95@gmail.com (I.G.); adrialhdez@biomat.uh.cu (A.A.); 2 National Institute of Technology, Rio de Janeiro-RJ 20081-312, Brazil; alexandre.antunes@int.gov.br (A.A.R.); varella_marize@yahoo.com.br (M.V.d.O.); 3 CUCEI, University of Guadalajara, Jalisco 44430, Mexico; lalomendizabal@gmail.com; 4 Experimental Trauma Surgery, Dept. Trauma Surgery, School of Medicine, Klinikum rechts der Isar, Technical University of Munich, 81675 Munich, Germany; patrina.poh@tum.de (P.P.); martijn.vangriensven@tum.de (M.v.G.)

**Keywords:** bone tissue engineering, scaffolds, hydroxyapatite, β-tricalcium phosphate, biphasic calcium phosphates

## Abstract

Ceramic materials mimic the mineral composition of native bone and feature osteoconductive properties; they are therefore used to regenerate bone tissue. Much research focuses on increasing the porosity and pore interconnectivity of ceramic scaffolds to increase osteoconductivity, cell migration and cell-cell interaction. We aimed to fabricate biocompatible 3D-scaffolds featuring macro- and microporous calcium phosphates with high pore interconnection. Nanoparticles of hydroxyapatite (HA) and calcium deficient hydroxyapatite (CDHA) were synthesized by wet chemical precipitation. Scaffolds were produced from them by the replication polymeric foam technique. Solid content and sintering temperature were varied. Nanoparticles and scaffolds were characterized regarding morphology, chemical and mineral composition, porosity and mechanical properties. Biocompatibility, cell attachment and distribution were evaluated in vitro with human adipose mesenchymal stem cells. Scaffolds with total porosity of 71%–87%, pores in the range of 280–550 µm and connectivity density up to 43 mm^−3^ were obtained. Smaller pore sizes were obtained at higher sintering temperature. High solid content resulted in a decrease of total porosity but increased interconnectivity. Scaffolds 50HA/50β-TCP featured superior interconnectivity and mechanical properties. They were bioactive and biocompatible. High HA solid content (40 wt.%) in the HA pure scaffolds was negative for cell viability and proliferation, while in the 50HA/50β-TCP composite scaffolds it resulted more biocompatible.

## 1. Introduction

Large bone defects appear with frequency in orthopedic clinical practice as result of trauma, infections or tumors [1]. In these cases, the native regeneration capacity of the injured tissue may be compromised. Different bone graft substitutes have been used for decades to replace and/or regenerate bone tissue with encouraging clinical results. This is particularly true in the case of ceramic materials [2,3,4,5].

Scaffolds for bone replacement are 3D structures that provide an appropriate architecture and environment for bone tissue regeneration whose composition may be tailored to mimic the mineral composition of the native bone. They serve as a template for cell interactions and the formation of bone-extracellular matrix that offers structural support to the newly formed tissue [6]. To perform this assignment, they must fulfill certain requirements in regard to their biological, mechanical and structural characteristics.

Biological features include biocompatibility, biodegradability and absence of toxic effects [7]. They have to support cell attachment, cell survival, proliferation and/or migration [8,9,10]. In some cases, these materials may act as carriers for growth factors, antibiotics and gene therapy [10]. In addition, they should stimulate vascularization to provide nutrient supply to the new tissue [11,12]. Biodegradability assures the elimination of the implant as the bone regeneration process advances. As a result, newly formed tissue will entirely colonize the lesion [8].

The mechanical properties of scaffolds should match the properties of the host tissue. This will reduce complications during the bone healing process [9,11]. Structural requests are a high degree of porosity with an appropriate interconnectivity for cell accommodation, growth, proliferation and migration. In addition, this will allow for nutrients flow, waste removal, vascularization and spatial organization [11]. The interlocking between scaffolds and surrounding tissues increases with the osteointegration process. However, not only the chemical nature of a scaffold influences bone ingrowth. Structural aspects such as porosity, pore size, shape and interconnectivity, as well as topography, also play a crucial role for in vivo bone ingrowth and tissue vascularization [12].

Despite copious efforts to clarify the link between the architecture and biological response of bone graft substitutes, this problematic is still not clear [13]. In vitro reports often do not match with in vivo findings [6]. Karageorgiou et al. concluded that in vitro, lower porosity stimulates osteogenesis by suppressing cell proliferation and forcing cell aggregation. In contrast, in vivo, higher porosity and pore size resulted in greater bone ingrowth [6]. It is widely accepted that macropores, i.e., pores in the range of 100 to 150 μm, are essential for both bone ingrowth and angiogenesis. However, pores of 50 μm in size have been described as sufficient for osteoconduction [14]. Higher pore sizes, i.e., between 200 and 500 μm, are desirable for the colonization of osteoblasts, vascular ingrowth and the deposition of new bone [14]. In contrast, micropores, i.e., pores typically in the range 0.1–10 µm, have been reported to support osteointegration in vivo. Lan Levengood et al. reported the colonization of such micropores in biphasic calcium phosphate scaffolds by osteogenic cells while osteocytes were embedded into the scaffold matrix after implantation into pig mandibular defects [15]. Recently, Bohner et al. [16] also demonstrated the relevance of such small pores for bone ingrowth in an ovine model with implanted porous β-tricalcium phosphate cylinders. The authors reported ingrowth of mineralized tissue in pores as small as 1 µm at 2–24 weeks post-implantation. The mineralized tissue formed was characterized by the presence of calcium phosphate and collagen as well as interconnected cells [16]. Similar to surface roughness, microporosity is also reported to affect the expression of cell adhesion proteins [17]. Moreover, high degrees of microporosity regulate the scaffold degradation rate as a result of the higher superficial area of contact. It also influences the mechanical properties and it may modulate the levels of calcium and phosphate ions, which in turn has an impact on the osteoblast viability [18], commitment [19] and maturation [20].

An additional problem is given by the application of identical bone graft substitutes and scaffolds to replace a great diversity of bone types. Different bones in the skeleton feature different architecture, vascularization, or mechanical requirements, which demand almost a specific internal structure for better osteointegration. Unfortunately, designing bone scaffolds to exactly and precisely match the native bone architecture is generally not feasible [13]. Additionally, the architecture of biodegradable scaffolds is variable as a consequence of the degradation process and the degradation by-products may modify the biological responses. Finally, the osteointegration process of a material depends not only on the material properties, but also on patient-related aspects such as age, gender, co-morbidities and genetics [13].

Calcium phosphates (CaPs) are the main constituents of the mineral phase of hard tissues in vertebrates [21]. In natural bone tissue, CaPs appear as ion-substituted calcium deficient hydroxyapatite commonly named “biological apatite”, which are always nanodimensional and nanocrystalline compounds [22]. For this reason, CaP biomaterials have generated great interest as bone tissue substitutes. For many years, increased attention has been given to developing new methodologies for obtaining and processing CaP bone substitutes [23]. CaP-based scaffolds, particularly hydroxyapatite (HA), β-tricalcium phosphate (β-TCP) and/or biphasic calcium phosphate (BCP) are among the most widely ceramic materials explored for bone tissue engineering applications [2,17,23,24]. CaPs assure biocompatibility and osteoconductive properties. The calcium and phosphate ions released by degradation induce an osteogenic response, contributing to the osteoinductivity of these materials [11], without presenting immunogenicity or toxic side effects [10]. Another recognized advantage of CaP-based scaffolds is the tailorable biodegradability when compared to other ceramics [25]. Unfortunately, the clinical application of ceramic-based scaffolds is limited due to their highly brittle nature, which restricts their use to low load-bearing applications [8,26].

In this context, the advances achieved in nanotechnology and the associated techniques have renewed interest in the synthesis and characterization of nanodimensional CaPs [22]. Nanocrystalline powders of HA [27,28] and β-TCP [29] have shown an improved sinterability and enhanced surface area; features that might improve the mechanical properties of nanosized HA materials [22]. Furthermore, from a biological point of view, nanodimensional CaPs are expected to feature better biocompatibility and bioactivity compared to coarser crystals [22,26]. For all these reasons, nanostructured ceramic scaffolds fabricated from nanosized CaP particles promise a larger potential with respect to micron-sized CaP materials.

In this work, the main objective was to fabricate scaffolds featuring macro- and microporous calcium phosphates with a high degree of interconnection, such as ceramic foams, and thereby mimic cancellous bone as best as possible. Therefore, nanoparticles of hydroxyapatite (HA) and poorly crystalline calcium deficient hydroxyapatite (CDHA) as a precursor of β-tricalcium phosphate (β-TCP) were synthesized by the wet chemical precipitation method. The produced nanoparticles were used as source for the development of biocompatible, highly porous 3D scaffolds. The CaP-based scaffolds were prepared by the replication polymeric foam technique with different experimental conditions. The influence of the processing parameters in the final scaffold architecture, i.e., pore size, porosity and interconnectivity, was evaluated. Bioactivity and mechanical strength were also explored. Furthermore, the biocompatibility of the scaffolds was studied in vitro by culturing human adipose mesenchymal stem cells (hAMSCs) for a period up to 14 days. These cells were chosen as a future goal is to use the scaffolds for supporting progenitor cells in bone engineering. Thus, the scaffolds should support the viability of these cells as a first pre-requisite. Cell viability was evaluated by lactate dehydrogenase (LDH) while cell proliferation was evaluated by PicoGreen assay. In order to gain insight regarding cell attachment and distribution inside the scaffolds, scanning electron microscopy was performed at 14 days post-seeding.

## 2. Results

### 2.1. Calcium Phosphate Particles Characterization

Two types of CaP powders were synthesized by means of the wet precipitation method. Their features were evaluated using different analytic techniques.

#### 2.1.1. Size and Morphology

**Figure 1:**
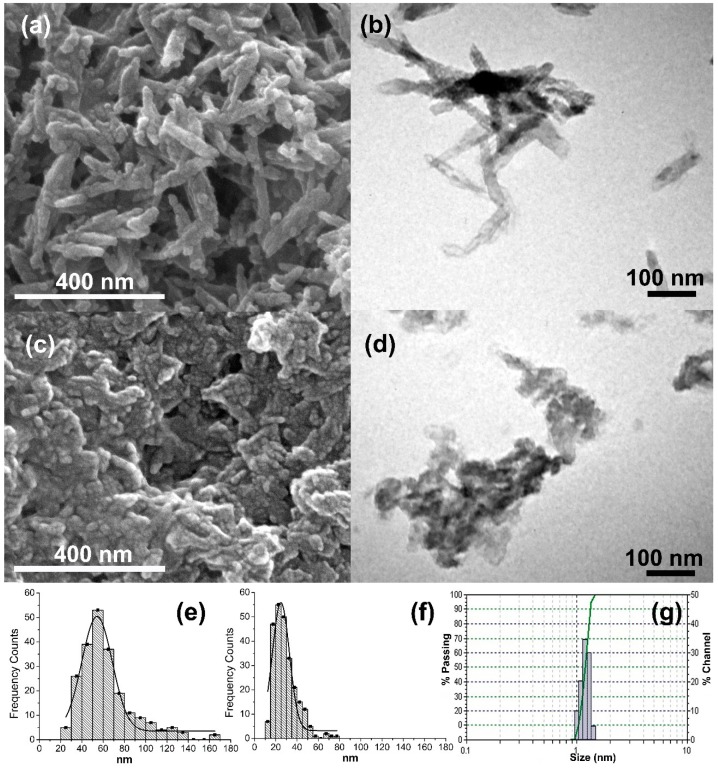
Morphological and dimensional characterization of HA (**a,b,e,g**) and CDHA (**c,d,f**) particles synthesized. (**a**,**c**) FESEM micrographs; (**b**,**d**) TEM micrographs and (**e**,**f**) size particle distributions calculated by FESEM image processing; (**g**) size particle distribution of nanoHA particles synthesized by Dynamic Light Scattering (DLS). Particle size illustrated in (**e**,**f**), and (**g**) correspond to the particles’ diameter.

The morphology and dimensions of HA and CDHA particles were observed by field emission scanning electron microscopy (FESEM) and transmission electron microscopy (TEM). Representative micrographs are shown in Figure 1. In both cases, nanodimensional particles were obtained. HA particles were in form of nanoneedles (63.5 ± 25.2 nm in length) (Figure 1a,b) while CDHA particles had irregular forms (28.6 ± 10.9 nm in diameter) (Figure 1c,d).

A detailed observation of the micrographs indicated that the nanoparticles were composed of even smaller particles. Moreover, both powders formed soft agglomerates easily. Dynamic light scattering (DLS) confirmed that the original particles were small, with diameter sizes near 1 nm each (Figure 1g) that quickly agglomerated to form more stable structures. Brunauer–Emmett–Teller (BET) surface measurements showed high surface area for both nanoparticles’ powders: 93.03 m^2^/g for HA and 55.48 m^2^/g for CDHA particles.

#### 2.1.2. Chemical and Mineral Composition

**Figure 2:**
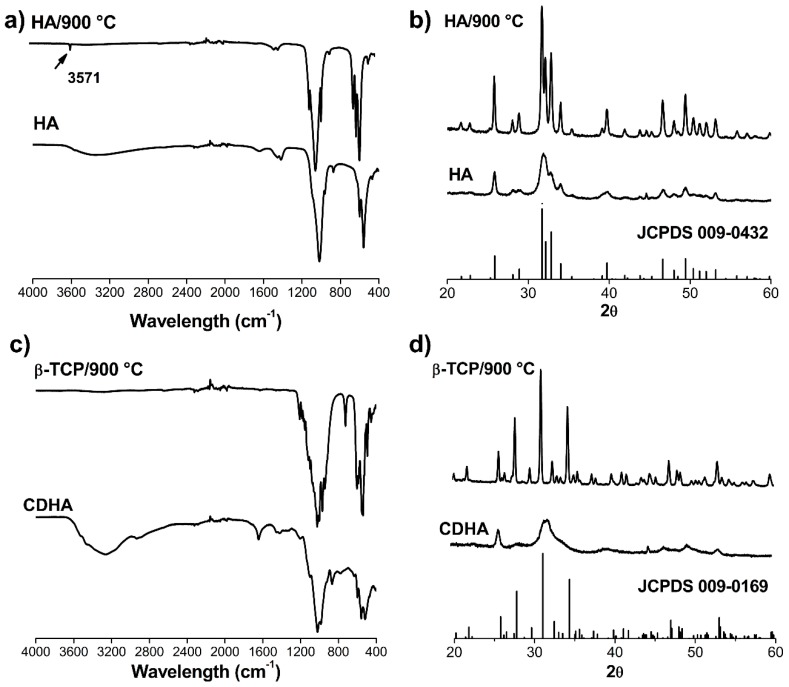
Chemical and crystallographic characterization of hydroxyapatite (HA) and poorly crystalline calcium deficient hydroxyapatite (CDHA) particles synthesized and heat-treated at 900 °C. (**a**,**c**) FTIR spectra; (**b**,**d**) XRD patterns.

Fourier transformed infrared (FTIR) spectra and X-Ray diffraction (XRD) patterns (Figure 2) of the obtained HA and CDHA powders revealed poor crystallinity. In the HA FTIR spectrum (Figure 2a), a typical broad band centered at approximately 3300 cm^−1^ was observed that can be associated with remaining water. Furthermore, bands present at 865, 1419 and 1464 cm^−1^ can be related to the presence of CO_3_^2−^. For more accurate identification, powder samples received a heat treatment at 900 °C and the bands at 560 and 606 cm^−1^ corresponding to υ_2_(PO_4_^3−^), 960 cm^−1^ to υ_1_(PO_4_^3−^), and 1068, 1095 cm^−1^ to υ_3_(PO_4_^3−^) were clearly assigned. In addition, the FTIR for the calcined HA showed a typical sharp vibration band derived from hydroxyl ions at 3571 cm^−1^ that can be attributed to the structural OH^-^ ions in crystalline hydroxyapatite: Ca_10_(PO_4_)_6_(OH)_2_.

Figure 2c showed the FTIR of the CaP powder synthesized with a 1.5 Ca/P ratio. In the literature, it is reported that synthetic amorphous calcium phosphate (ACP) after several hours is converted in poorly crystalline CDHA [30]. For this CaP powder, the FTIR bands of absorbed water (around 3300 cm^−1^) and bands associated with PO_4_^3−^ groups (1000–1100 cm^−1^) were detected. In addition, the band at 869 cm^−1^ is due to the P-(OH) stretch of the HPO_4_^2−^ ion and the band at 1644 cm^−1^ can be associated with the presence of some CO_3_^2−^ ions or water [31]. The FTIR characterization indicated the presence of CDHA. For calcined CDHA (converted to β-TCP), the υ_3_(PO_4_^3−^) (1107 and 1031 cm^−1^) and υ_4_(PO_4_^3−^) (604 and 544 cm^−1^) bands were detected.

XRD patterns indicated wide reflections for both synthesized materials. This may be due to the low crystallinity degree of the samples. Refined reflections of the heated materials were in good agreement with the Joint Committee on Power Diffraction Standards (JCPDS) cards 00-009-0432 (Figure 2b) and 00-009-169 (Figure 2d) for HA and whitlockite (β-TCP), respectively. No other calcium phosphate crystalline phases were detected in the respective patterns corroborating the chemical and mineral purity of the obtained powders.

### 2.2. Scaffolds Characterizations

**Figure 3:**
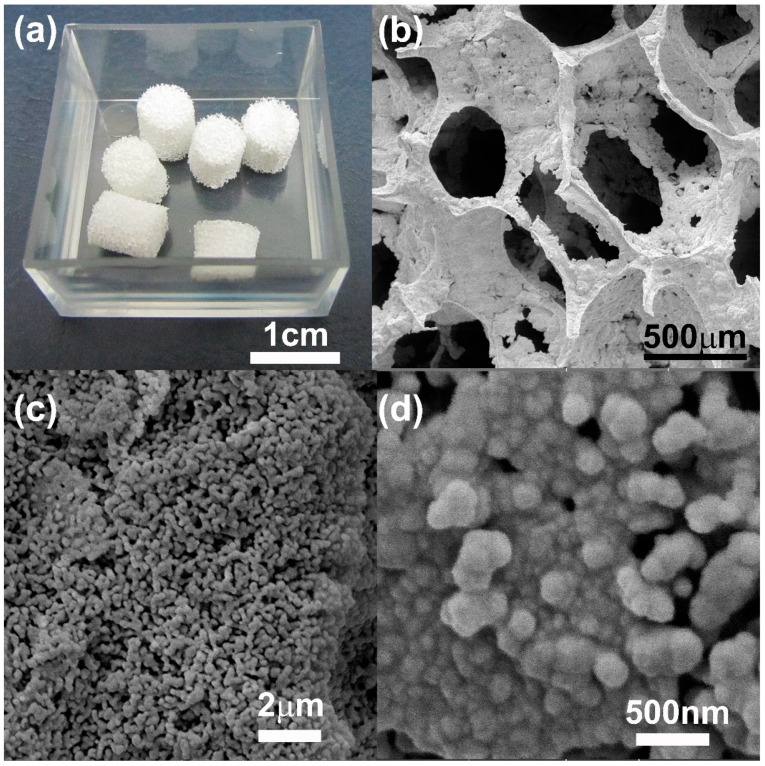
(**a**) 100HA/40 wt.%/1100 °C scaffolds as obtained are shown as representative image of all the fabricated scaffolds; (**b**–**d**) FESEM micrographs are shown of 100HA/20 wt.%/800 °C scaffolds at different magnifications.

In this work, different CaP scaffolds were fabricated using the pure HA or CDHA nanoparticles previously synthesized. The replication polyurethane foam technique with different processing conditions was used as fabrication method. In all cases, scaffolds with internal highly interconnected 3D porous architecture were obtained. The appearance of the obtained scaffolds can be concluded from Figure 3a.

#### 2.2.1. Physicochemical, Morphological and Mechanical Characterization of Scaffolds

The initial experimental processing condition tested to prepare the CaP scaffolds was 20 wt.% of solid content and sintering temperature of 800 °C with 6 h of dwelling. The aim was to maintain a topography characterized by lower dimensions in the ceramic grains. Furthermore, the goal was to determine the variation of grain size with the processing conditions.

At this lower temperature, i.e., 800 °C, the grain size in the scaffold walls were in the nanometer range (287 ± 99 nm). The size of the scaffolds’ pores in diameter was of 460 ± 70 µm. Structures with highly interconnected macroporosity were achieved. These scaffolds (20 wt.% of solid content) were difficult to manipulate; they resulted brittle and fragile. Figure 3b illustrates how thin the pores’ walls are, which is responsible for the overall fragility of the scaffolds. In addition, macroporosity, microporosity as well as nanoporosity were detected in the scaffolds structure (Figure 3c,d). The increase of solid content, from 20 wt.% to 40 wt.%, greatly improved the manipulative properties. Scaffolds were robust and structurally stable. However, because of the high volume of particles caused by its nanodimensional sizes, increase of the solid contents from 20 wt.% to 40 wt.% substantially reduced the porosity of the scaffolds (Table 1).

Increasing the sintering temperature up to 1200 °C improved the manipulative properties of the scaffolds. Thus, similar to the solid content, the sintering temperature may have a positive effect on the mechanical properties of the scaffolds.

**Figure 4:**
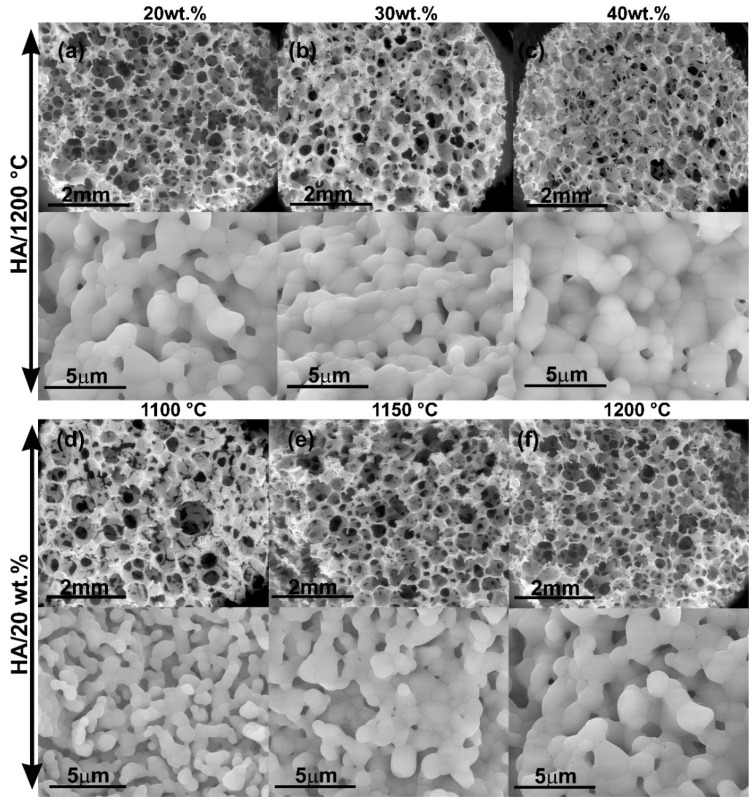
FESEM micrographs of scaffolds of HA fabricated with sintering temperature of 1200 °C and using different slurry solid content. (**a**) 20 wt.%HA, (**b**) 30 wt.%HA, (**c**) 40 wt.%HA; FESEM micrographs of scaffolds of HA with 20 wt.% of solid content and fabricated at different sintering temperature: (**d**) 1100 °C, (**e**) 1150 °C, (**f**) 1200 °C.

Figure 4a–c shows the interconnected structure of the pure HA scaffolds obtained at the same sintering temperature (1200 °C) varying the solid content in the slurry. The increase of the solid content did not affect statistically neither the pore size nor the dimensions of the ceramic grains in the walls of the scaffolds as determined by FESEM (Table 1). The obtained pore dimensions were appropriate for the further colonization and survival of bone cells. However, Micro Computed Tomography (µCT) analysis showed a direct influence of the solid content on the total porosity and the interconnection density of the scaffolds (Table 1). Increasing particle content from 20 wt.% to 40 wt.% decreases the total porosity from 87% to 75%, but increases the density of pore interconnection from 18 mm^−3^ to 26 mm^−3^.

Figure 4d–f shows micrographs of scaffolds of HA fabricated with 20 wt.% of slurry solid content at different sintering temperature. An increase in the sintering temperature from 1100 °C to 1200 °C resulted in a decrease of the pore size and interconnection while increasing the grain size and total porosity of the scaffolds (Table 1). However, all scaffolds maintained low manipulative properties. For this reason, simultaneous increments of slurry solid content and sintering temperature were required and were performed in the subsequent experiments.

**Figure 5:**
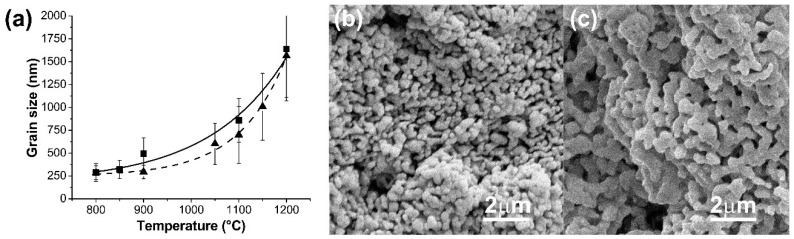
(**a**) Dependence between grain size and sintering temperature for scaffolds of HA: -▲-20 wt.%, Dash line; -■- 40wt.% Full line. (**b**,**c**) FESEM micrographs of HA/β-TCP scaffolds (**b**) 80HA/20β-TCP, (**c**) 20HA/80β-TCP.

Figure 5a summarizes the relationship between the mean grain size and the sintering temperature for samples with 20 wt.% or 40 wt.% of slurry solid content of HA. No statistical differences (*p* > 0.05) were detected between the mean grain size as a function of the slurry solid content. However, a significant increase in grain size was detected with increasing sintering temperature passing from nanostructured topography at 800 °C to micrometrics grain dimension at the scaffold surface.

It is well known that HA is a biomaterial relatively insoluble in vivo. By contrast, β-TCP shows good bioreabsorption in vivo [32]. With the purpose of granting a certain degree of biodegradation to the 3D structures and trying to adjust the kinetics of biodegradation to the rate of formation of new bone without abrupt deterioration of the final mechanical properties of the scaffolds, several 40 wt.% of biphasic HA/β-TCP systems were also prepared. Compositional changes also influenced the superficial topography of the scaffolds. This was observed to be a function of the HA/β-TCP ratio. Figure 5b–c shows the topography of the scaffold walls for different HA/β-TCP samples sintered at 900 °C. The mean grain size for 80HA/20β-TCP scaffold was 330 ± 50 nm (Figure 5b), while the grain size values for 20HA/80β-TCP scaffold were 620 ± 140 nm (Figure 5c).

**Figure 6:**
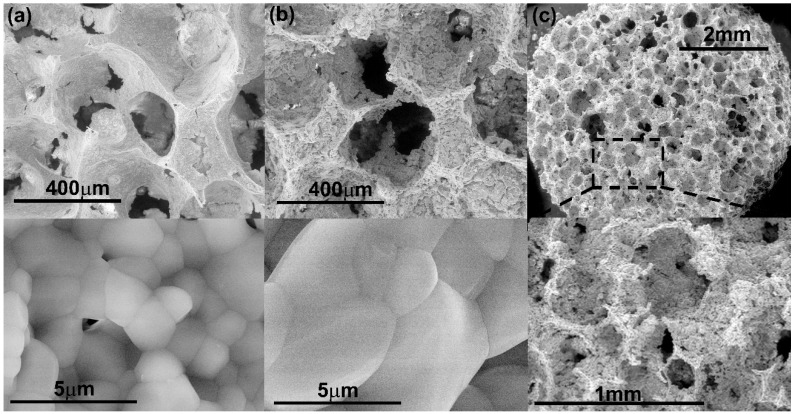
Micrographs of the structure of scaffolds with 40 wt.% of solid content and fabricated at 1200 °C. (**a**) HA; (**b**) and (**c**) β-TCP.

The chemical composition of scaffolds also impacted pore size, grain size, total porosity and the interconnection density of the scaffolds. To illustrate this, Figure 6 shows micrographs of the scaffolds made of 40 wt.% of pure HA or pure β-TCP at higher sintering temperature (1200 °C). Firstly, it is clearly observed that the grain size of β-TCP scaffolds (Figure 6b) was higher in comparison with the grain size of HA scaffolds (Figure 6a). According to the heating microscopy results, nanoparticles of the CDHA powders had a lower sintering beginning temperature (839 °C) in comparison with those of the HA nanoparticles (916 °C). On the other hand, pure β-TCP scaffolds made with 40 wt.% of CDHA solid content in the slurry gave rise to significantly closed pores at the scaffold’s surface (Figure 6c).

**Figure 7:**
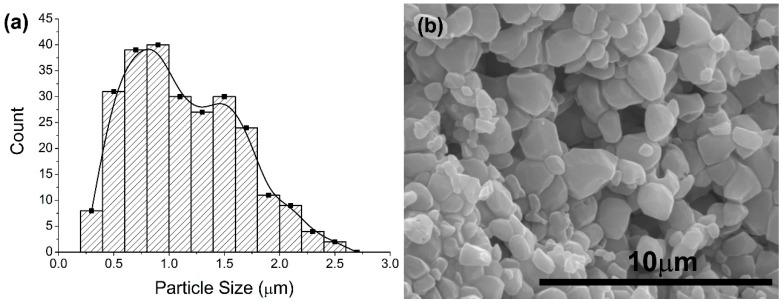
Biphasic scaffolds (50HA/50β-TCP) obtained with 40 wt.% of slurry solid content and 1200 °C. (**a**) Histogram of grain size. (**b**) Micrographs of the structure.

**Figure 8:**
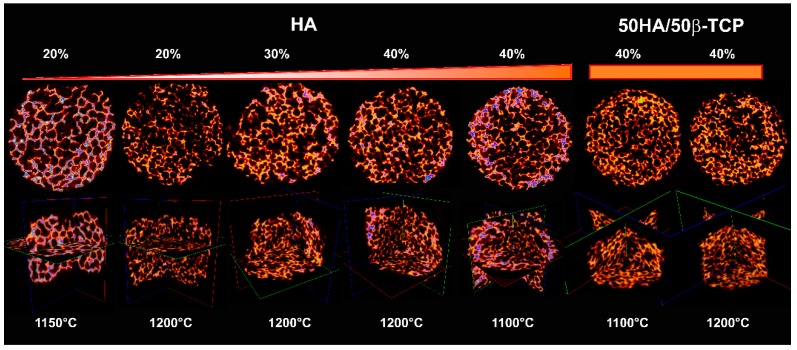
µCT reconstruction of pure HA and 50HA/50β-TCP biphasic scaffolds.

The 50HA/50-βTCP scaffolds were superior based on the significant differences in properties such as mechanical properties or porous interconnectivity. Therefore, these samples were used for the subsequent biological tests (Figure 7 and Figure 8). Thus, Figure 7a shows the histogram for grain sizes at the scaffold wall for 50HA/50β-TCP samples sintered at 1200 °C. A bimodal size distribution is clearly observed which corresponds to the presence of grains of HA (with lower sizes) and grains of β-TCP (with higher sizes) (Figure 7b). Heating microscopy analysis indicated that the beginning sintering temperature of 50HA/50β-TCP samples was 902 °C. The variation of scaffold composition from pure HA to 50HA/50β-TCP did not affect the total porosity of samples but increased in almost twice the pore interconnection of the 3D structure (Table 1). Differences in sintering temperature (1100, 1150 and 1200 °C) exhibited the same effect.

Figure 8 summarizes the µCT characterization. Micrographs clearly revealed the high porosity of all samples and the interconnected nature of the 3D structures obtained.

#### 2.2.2. Chemical and Crystallographic Characterizations

**Figure 9:**
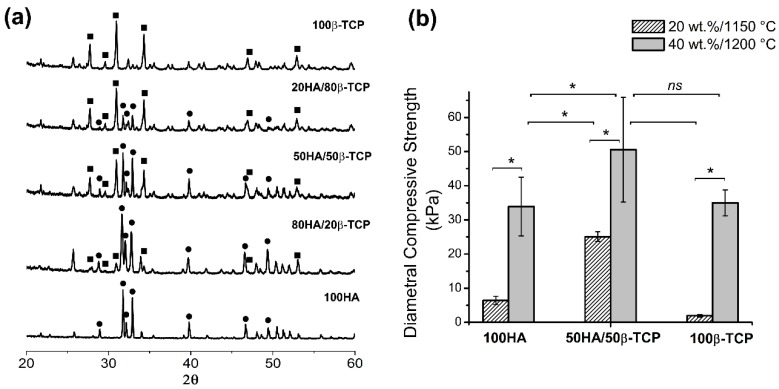
Characterization of scaffolds made of pure HA, pure β-TCP or biphasic HA/β-TCP. (**a**) XRD patterns of scaffolds with 40 wt.% fabricated at 1200 °C, -■-β-TCP, -●- HA; (**b**) Diametral compression strength at different conditions. *p < 0.05.

Figure 9a shows XRD patterns of scaffolds prepared with 40 wt.% of slurry, sintered during 6 h at 1200 °C but considering different chemical compositions. Scaffolds of pure HA and pure β-TCP fully matched the HA card (JCPDS 00-009-432) and β-TCP card (JCPDS 00-009-169). Scaffolds made with other ratios, i.e., 80/20, 50/50 and 20/80 of HA/β-TCP only showed a combination of the HA and β-TCP phases without the presence of other crystalline phases.

Figure 9b shows the values obtained for the diametral compression strength (DCS) test. In general, the resistance was low (in the order of kPa). Scaffolds of pure phases with less slurry solid content (20 wt.%) and lower sintering temperature (1150 °C) had the lowest values by DCS, i.e., 1.5 ± 0.4 kPa for β-TCP and 6.4 ± 1.2 kPa for HA. Scaffolds of 50HA/50β-TCP with the higher solid content of 40 wt.% and sintering temperature of 1200 °C showed the highest strength in diametral compression assays at 50.5 ± 15.3 kPa.

#### 2.2.3. Bioactivity Test

**Figure 10:**
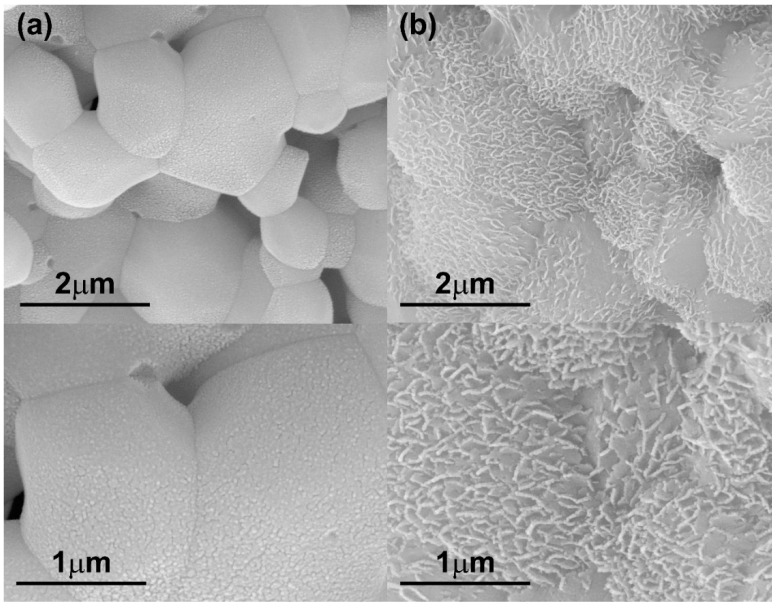
FESEM micrographs of HA scaffolds after 30 days of soaking in SBF at different magnification. The scaffolds were sintered at 1200 °C and using (**a**) 20 wt.% of slurry solid content, (**b**) 40 wt.% of slurry solid content.

The bioactivity tests of the HA scaffolds showed that morphological changes occurred earlier in scaffolds with higher solid content. Figure 10 shows that after 30 days of immersion in simulated body fluid (SBF), samples with HA/20 wt.% only had an incipient change on the surface scaffolds morphology while samples with HA/40 wt.% presented morphological changes in a greater surface extension and in greater magnitude.

**Figure 11:**
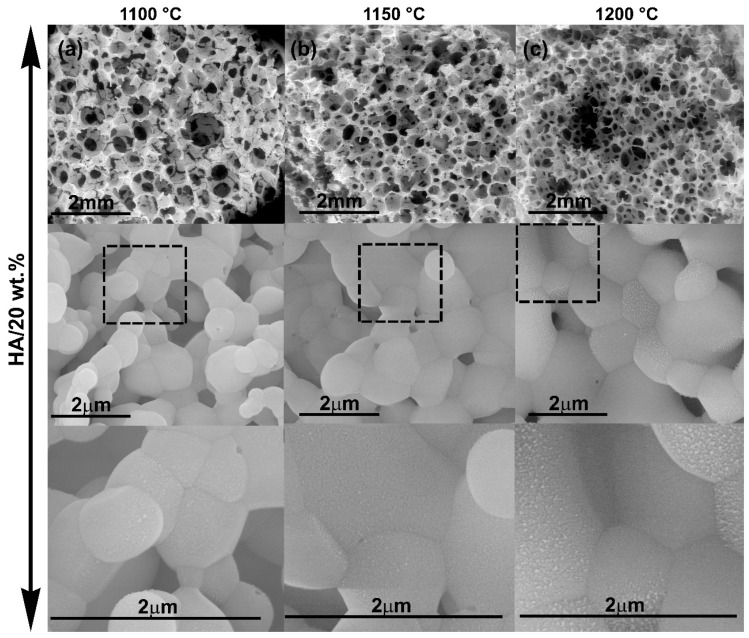
FESEM micrographs of HA scaffolds after 30 days of soaking in SBF at different magnification. The scaffolds had 20 wt.% of slurry solid content and were sintered at different temperatures (**a**) 1100 °C, (**b**) 1150 °C and (**c**) 1200 °C.

Figure 11 highlights the effect of sintering temperature on sample bioactivity. The increase in sintering temperature from 1100 °C to 1200 °C resulted in increased bioactivity.

#### 2.2.4. Biocompatibility Evaluation

**Figure 12:**
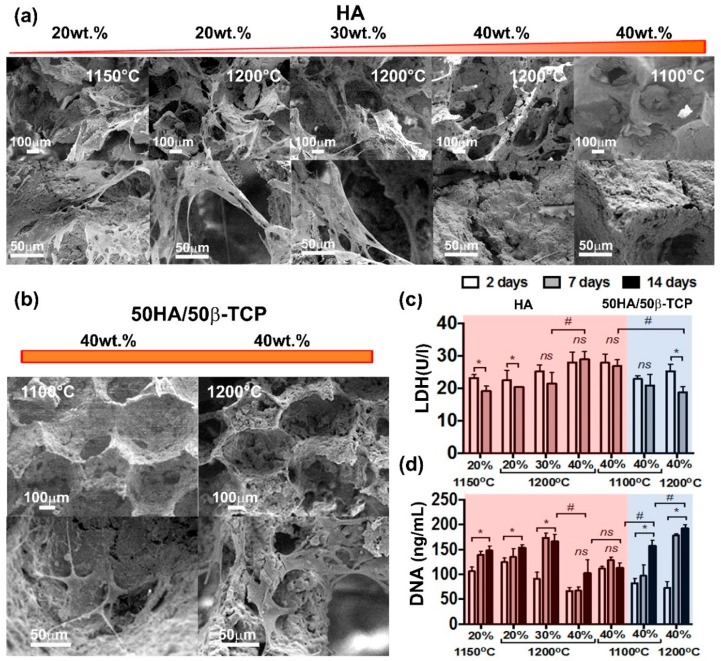
In vitro biocompatibility assessment with hAMSCs. (**a**,**b**) SEM observations at 14 days after cell seeding; (**c**) Cell viability by LDH; (**d**) Cell proliferation by PicoGreen assay.

The biocompatibility of the scaffolds was evaluated in vitro by culturing human adipose mesenchymal stem cells (hAMSCs) for a period up to 14 days (Figure 12). Possible toxicity of produced scaffolds was assessed by means of LDH activity released upon cell death (Figure 12c). For scaffolds made of HA, an increase in the solid content resulted in increasing activity of LDH. This was statistically significant for scaffolds with a solid content greater than 30 wt.% (Figure 12c). Interestingly, the sintering temperature did not show any significant effect on cell viability. Scaffolds of 50HA/50β-TCP were significantly less toxic than their HA counterpart. Lower LDH activities were detected in the 50HA/50β-TCP scaffold groups. Remarkably, in scaffolds with the highest solid content, i.e., 40 wt.%, 50HA/50β-TCP scaffolds resulted in significantly less LDH activity than HA scaffolds. These results elucidate the effect of the composition on the toxicity of the produced scaffolds.

Increasing the HA content to 40 wt.% also resulted in a significant decrease in cell proliferation (Figure 12d). Also, in line with the LDH results, 50HA/50β-TCP scaffolds obtained by using 40 wt.% of solid content showed significantly higher cell proliferation than the HA scaffolds. SEM observation at 14 days after cell seeding supports the LDH and DNA results. Cells were observed in the HA scaffolds with 20 wt.% and 30 wt.%. (Figure 12a,b). In fact, in those scaffolds, cells were shown to attach and spread well on the surface, and on the pores. Conversely, negligible presence of cells could be concluded in the HA 40 wt.% scaffolds. This situation was reverted by utilizing 50HA/50β-TCP scaffolds. In 50HA/50β-TCP 40 wt.% scaffolds, cells could be identified inside the pores and attached to the scaffold surface.

## 3. Discussion

The characterization of synthetized calcium phosphate powders revealed that both HA and ACP (converted in poorly crystalline calcium deficient hydroxyapatite) particles were obtained with nanodimensional size distribution, in spite of the agglomeration in more stable structures that they conformed. The morphology was different for the two types of particles; HA particles appear as nanoneedles that is in agreement with reported literature [33]; while CDHA particles had irregular forms. In our study, we found that CDHA particles were smaller than HA particles and with a narrower distribution of sizes. Interestingly, the HA particles have a higher superficial area value in comparison with CDHA particles, i.e., BET_HA_ > BET_CDHA_. This could be explained by the structural features of the particles. In our study, particle dimensions correspond to the dimensions of the stable structures that spontaneously formed the original nanoparticles. However, these structures are conformed by agglomeration of smaller particles. The agglomerates that conform the HA particles may present high rugosity that in turn results in high superficial area values. The specific surface area values found in our study were in the order of the values reported in the literature, which included a wide range from 23.27 m^2^/g [34] to 103.05 m^2^/g [35].

FTIR spectra as well as the XRD patterns of the synthetized powders indicated low crystallinity for the calcium phosphate particles produced, in addition to a high chemical purity without the presence of other crystallographic phases in each material.

Scaffold characterization results demonstrated the presence of 3D pore-architecture with a high degree of interconnection for all compositions tested. According to the literature, the foam replication technique is an appropriate method to mimic trabecular bone architecture. Swain et al. fabricated pure HA scaffolds using the replication foam scheme [34]. They increased the slurry solid loading from 30 to 50 wt.% to prepare scaffolds with apparent porosity values from 69% to 56%, but not all compositions showed interconnectivity [34]. Cunningham et al. compared the architecture of 3D hydroxyapatite scaffolds developed via replication of synthetic polyurethane foams vs natural marine sponges using a high solid content (80 wt.%) [36]. The use of synthetic polymeric foam yielded scaffolds with pore sizes ranging from 50–1000 μm (average pore size 577 μm), 99.99% pore interconnectivity and average compressive strengths of 2.46 ± 1.43 MPa [36]. Otherwise, Wang et al. prepared biphasic CaPs scaffolds using a slurry concentration between 40 wt.% to 50 wt.% obtaining highly interconnected microporous structures with open macropores ranging from 500 to 900 μm, grain sizes in a range of 0.5–2 μm and a compressive strength of 1.11 ± 0.1 MPa [37].

In our study, FESEM observations showed that the wall topography of scaffolds (ceramic grains) changed from nanosized to microsized in a straight relationship with sintering conditions. Several processes have been reported to take place during sintering of CaP powders [2]. The CDHA powder is converted into β-TCP. In addition, to the chemical changes, there is an increase in crystal size and a decrease in the specific surface area accompanied by an increase in the mechanical strength [38,39]. For HA compacted powders, it is reported that sintering below 1000 °C leads to initial particle coalescence, with little or no densification and a significant loss of surface area or porosity [2]. On the other hand, processing at higher temperatures is not recommended, because this may lead to exaggerated grain growth and decomposition of HA that becomes unstable at temperatures exceeding 1250–1300 °C [40].

It is argued that the small dimension of wall grains, as well as microporosity, provides both a greater surface area for protein adsorption as well as an increased ionic solubility accelerating scaffold biodegradation [2]. According to Lan Levengood et al., a multiscale osteointegration in CaP-based scaffolds occurs through the combination of open macropores and interconnected micropores [15] whereas Rustom et al. demonstrated that micropores induce capillarity that enhances bone distribution in vivo in biphasic calcium phosphate scaffolds [41]. For all these reasons, it is necessary to maintain a compromise between all features of the internal scaffold structure for better biological performance and preserving an adequate final mechanical strength.

In our work, micrographs of samples sintered at 800, 850 and 900 °C revealed a topography with a mean of grain sizes between 250–500 nm for pure HA scaffolds, whereas samples at higher sintering temperatures evidenced a micrometric distribution of grain sizes without influence of the solid content of the slurry until 40 wt.%. In the case of HA/β-TCP samples processed at 900 °C, a moderate increase in grain size was detected in comparison with pure HA sintered at the same temperature. This is attributable to the increase of β-TCP grain size, which had a lower beginning sintering temperature (839 °C). According to the literature, ACP powders during their transformation to β-TCP presented a linear shrinkage (associated with the beginning of sintering process) around 860–870 °C [42]. In our work, the beginning sintering temperature values detected for ACP (converted to CDHA) particles were lower given the nanodimensional nature of the synthetized particles. For the same reason, HA/β-TCP samples presented a bimodal distribution of grain sizes at higher sintering temperature.

As mentioned before, the scaffolds porosity facilitates the mechanical fixation of the implants, because it provides surface sites for chemical bonding between the bioceramics and bones [43]. In addition, pore size is an important property for surface and space for cell adhesion and bone ingrowth, while pore interconnection offers the way for cell distribution and migration and in vivo to blood vessel formation needed for osteointegration processes [6,44,45]. In this work, the scaffold porosities obtained were ranged between 71%–87%, which is appropriate for desired bone ingrowth. All obtained scaffolds presented macroporosity with pore sizes around 300 μm or larger. This has been recommended in the reported literature in order to enhanced new bone formation and the formation of capillaries [6,14]. An increase in sintering temperature as well as the inclusion of β-TCP in the scaffolds’ composition decreased the mean of macropore size as well as a certain tendency to increase connectivity density. On the other hand, our results indicated that the increase of solid content in the slurry causes a reduction in microporosity in the scaffold walls. However, similar macropore sizes were maintained and a slight increase in interconnectivity was obtained.

No additive remains inherent to the obtaining processes were detected in agreement with other reports [37]. In addition, sintering treatment until 1200 °C did not produce new crystalline phases in the materials, indicating the adequate Ca/P ratio of each nanoparticle synthetized.

The processing parameters used in the replication foam technique, i.e., solid content of the slurry, sintering temperature and chemical composition, had a direct impact on the mechanical properties of the porous ceramic pieces. Diametral compression strength values as an indirect measure of tensile strength have been reported before [46] and in our study they were in the order of kPa. According to the literature, hydroxyapatite scaffolds obtained by replication of the sponges with high porosity (82%–86%) showed low compressive strength values in a range of 0.2–0.4 MPa [47]. The higher solid content and sintering temperature made a significant increase in the final mechanical properties of the scaffolds, which in first instance improved the manipulative properties and lastly it may impact the mechanical bone compatibility. The scaffold’s mechanical behavior revealed a contribution of all features that characterize its 3D structure: total porosity, pore interconnectivity, size pore distribution, grain size, and of course, chemical nature of components. The higher slurry solid content (40 wt.%) and the higher temperature of sintering (1200 °C) could be related to the higher diametral compression strength (50.5 ± 15.3 kPa) presented in 50HA/50β-TCP scaffolds. In comparison with pure HA scaffolds obtained by similar processing parameters, the total porosity was similar, however, the range of the macropores of HA/β-TCP scaffolds was narrow in comparison with HA scaffolds. In addition, HA/β-TCP scaffolds were constituted by particles with different chemical nature and size, resulting in improved mechanical properties, as others report [48]. Unfortunately, diametral compressive strength values for ceramic calcium phosphate scaffolds, foam type, with porosity, connectivity density and similar pore sizes that allow a comparison, are not available in the literature. In any case, the obtained mechanical property values for our developed scaffolds may restrict their application to low-load bearing medical applications.

As expected, all compositions tested, 20 wt.% to 40 wt.% of HA slurry solid content showed surface morphological changes by soaking in SBF. The greater extension of deposition corresponded with scaffolds having higher amount of HA (calcium and phosphorous) per SBF volume. The increase in sintering temperature increased the in vitro scaffold bioactive response associated with the increment of total porosity and a better flow of SBF fluid.

Interestingly, a higher amount of HA in our scaffolds resulted in slightly toxicity to hAMSCs and negatively impacted cell proliferation. Similar observations were reported by Kamal et al. for a nanoHA paste scaffold [49]. The authors reported changes in cell morphology and impairment of cell attachment. A proliferation inhibition value of 40% was reported for those nanoHA scaffolds. Unfortunately, no HA solid content was described for the tested material [49].

Huang et al. recently reported a slight decrease on cell viability using hAMSCs on scaffolds with HA solid content of 20 wt.% when compared with their 10 wt.% analogue [48]. Nevertheless, no statistical differences could be concluded. Pawelec et al. also reported in a recent study that osteoblast-like cells proliferated well on collagen-HA scaffolds, reaching the highest proliferation rates in 20 wt.% HA scaffolds [50]. The authors highlighted that cell proliferation clearly decreased by increasing the solid content to 40 wt.%. This observation from Pawelec et al. is in line with our results. The authors justified the decrement on cell viability and proliferation with the increased surface roughness in the 40 wt.% scaffolds. In our study, all HA scaffolds from 20 wt.% to 40 wt.% featured very similar macropore sizes between 330–340 µm. A pore size of 300 µm has been reported as the optimal balance between diffusion and surface area allowing best blood vessel infiltration and supporting mineralization for bone [50]. However, total porosity indeed decreased with the increase of HA solid content. This decrease in total porosity for the 40 wt.% HA may result in less access to the cells to the inner part of the scaffolds together with less nutrient diffusion, which in turn will explain the negative effect observed on cell viability and proliferation.

Huang et al. also reported that scaffolds with β-TCP in their composition were significantly more biocompatible than HA scaffolds alone [48]. This observation matches well our findings. In our study, 50HA/50β-TCP scaffolds showed a significant increase in cell viability and proliferation when compared to pure HA despite their higher solid content of 40 wt.%. This indicates the high biocompatibility that characterizes β-TCP materials.

## 4. Materials and Methods

### 4.1. Synthesis of Calcium Phosphate Nanoparticles

CaP particles were synthetized by the wet precipitated method. A suspension of calcium hydroxide (Ca(OH)_2_, p.a SPECTRUM, New Brunswick, NJ, USA) was used as calcium ion precursor and for PO_4_^3−^ ions a solution of phosphoric acid (H_3_PO_4_, p.a. TEDIA Company, Fairfield, OH, USA) was the phosphorous source.

Stoichiometric conditions were established to obtain:

- HA powders Ca_10_(PO_4_)_6_(OH)_2_, Ca/P ratio of 1.67,
- or ACP powders Ca_3_(PO_4_)_2_, Ca/P ratio of 1.5.

In the case of ACP synthesis, to facilitate the previous solubility of calcium hydroxide, 100 g of commercial sucrose was added. For both syntheses, i.e., HA and ACP, the calcium suspensions were stirred for 1 h before starting the acid drip at a rate of 4 mL/min. The stirring was maintained at 1200 rpm for the HA synthesis and 900 rpm for the ACP synthesis during the reaction time of and for 1 h more after finishing the experiment. The final pH of the HA reaction was adjusted to 10.5 using drops of NaOH (1M, p.a. MERCK, Haar, Germany). Both CaP suspensions obtained were left to rest overnight. Thereafter, the mother liquors were removed by decantation and the particles were abundantly washed at least for three times with double distilled water (ddH_2_O). Next, the suspensions were centrifuged (ROTINA 420R, HETTICH, Tuttlingen, Germany) and finally lyophilized (LS3000, TERRONI, São Carlos, Brazil).

### 4.2. Characterizations of CaP Nanoparticles

Dynamic light scattering (DLS) (Zetatrac Measurement Analyzer NPA152-31A, Microtrac, Montgomeryville, PA, USA) analysis was performed to characterize the size of the synthesized particles. A fraction sample of the CaP suspensions was collected before the centrifugation and drying step during the synthesis. The sample was diluted in ddH_2_O, and 10 µL were mixed with 1 mL of acetone. Next, the mixture was sonicated for 1 min just before performing the DLS measurement. The specific surface area of the powders was measured by the application of gravimetric nitrogen Brunauer–Emmett–Teller (BET) surface analysis technique, using a Micromeritics ASAP 2020 Surface Area and Porosity Analyser (Norcross, GA, USA) with the sample outgassed.

Field emission scanning electron microscopy (FESEM) Quanta FEG 450 (FEI Company, Hillsboro, OR, USA) micrographs were taken to characterize the morphology of the synthetized particles. The suspensions of particles were sonicated in ethanol, dried over carbon tape and sputtered with platinum (Pt) (Sputter Coater, K550X–EMITECH, Ashford, England) for FESEM observations. Particles size dimensions were measured using NIH ImageJ 1.39u software (National Institutes of Health, Bethesda, MD, USA). Also, the nanoparticles obtained were observed by transmission electron microscopy (TEM) (JEOL 1010, Peabody, MA, USA). In this case, the suspensions of particles were sonicated in ethanol and directly dried over TEM grids.

Infrared spectroscopy (FTIR, Spectrum^TM^ 100, Perkin Elmer, Waltham, MA, USA) was performed to evaluate the chemical composition of the powders. For this, 10 mg of each sample was mixed with KBr (IR spectroscopy quality, SIGMA-ALDRICH, St. Louis, MO, USA) and then pressed into translucent pellets for the measurement. Spectra between 4000–400 cm^−1^ ranges were obtained from co-addition of 30 scans.

Powder samples before and after heat treatment (900 °C) were manually pressed in cylindrical standard sample holders for X-ray diffraction (XRD) analyses. PANalytical X’Pert PRO MPD Alpha1 (The Netherlands) powder diffractometer in Bragg−Brentano θ/2θ was used to obtain XRD patterns. Cu-Kα1 radiation (λ = 1.5406 Å) was employed and the measuring was taken from 10 to 90° in 2θ with step size of 0.02°. XRD patterns were processed using the card database of the Joint Committee on Powder Diffraction Standards (JCPDS).

Heating microscopy was performed on pure HA, CDHA and to 50HA/50CDHA samples. For this, cylindrical specimens of around 30 mg with 3 mm in diameter and 3 mm in height were heated at 5 °C/min up to 1450 °C. During heating, a picture was taken every 3 min in the first part of the experiment and with a frequency every 3 s from 700 °C to the end.

### 4.3. Porous Scaffolds Preparation Technique

Lyophilized CaP particles were used to prepare the porous scaffolds through the replication polyurethane foam technique. The particles were suspended in polyvinyl alcohol aqueous solution (PAA) at 8 wt.% (${\bar{M}}_{w}$ = 106 000−110 000 g/mol) (SIGMA-ALDRICH, St. Louis, MO, USA). Ultrasound cycles of 5 min (Ultrasonic Probe Hielscher UP400S 24kHz, Germany) and mechanical agitation cycles of 10 min (Magnetic stirrer Marconi ACP 085/CT – N480D; Piracicaba, Brazil) were applied to the suspension to guarantee its homogeneity. Polyurethane foam templates were cut into a cylindrical shape (6 mm in diameter × 10 mm in height), washed abundantly with ddH_2_O, and dried at 100 °C for 2 h prior to the experiment. The prepared sponges were immersed in the slurries and gently compressed at least three times to remove the air and to allow the slurry penetration into the pores of the foams. The impregnated sponges were superficially cleaned with filter paper on all sides to remove excess slurry (without squeezing) and air sprayed for 10 s in order to open the external pores. Then, the sponges were dried at 37 °C for more than 24 h. Later, the green bodies received the appropriate thermal process for sample sinterization. The heat treatment was performed as follows: ramp up to 400 °C at 2 °C/min, held for 1 h to burn out the polyurethane foams, then ramped up to the sintering temperature at 4 °C/min with a dwell time of 6 h at this temperature to obtain the final ceramic scaffolds.

Table 2 (Part I) shows the general conditions and samples compositions prepared. Not all scaffolds were tested for all parameters; the best featured scaffolds were selected for subsequent analysis (Table 2, Part II). Pure 100 wt.% of HA or 100 wt.% of CDHA scaffolds were prepared in addition to biphasic scaffolds. The names of the samples conformed to the nature of particles/solids content in the slurry/sintering temperature.

### 4.4. Physicochemical, Morphological and Mechanical Characterization of Scaffolds

X-ray diffraction analyses were performed using pulverized scaffolds with different compositions and different sintering temperature (Table 1). The aim of XRD was the verification of the phase compositions on the resulting material. The equipment and operating conditions were similar to those described in the Section 4.2.

The microstructure of the resulting scaffolds was studied using Field Emission Scanning Electron Microscopy (FESEM, Quanta FEG, 450, Hillsboro, OR, USA). Thereby, pore size, morphology, distribution, and interconnection were evaluated. To make the scaffolds conductive, samples previously adhered to carbon tape were platinum (Pt) sputtered during 3 min using a Sputter Coater (K550X – EMITECH, Ashford, England) prior to the observations. Particles size and pore dimensions were measured using NIH ImageJ 1.39u software (National Institutes of Health, Bethesda, MD, USA). ANOVA statistical analysis was used to determine significant differences. A *p*-value of less than 0.05 was considered statistically significant.

Three-dimensional (3D) structure and scaffold architecture were evaluated by means of Micro Computed Tomography (µCT). The scaffolds were scanned using a Skyscan 1176 μCT (BRUKER, Kontich, Belgium) at 40kV with a voxel size of 9µm. Image reconstruction was performed using NRecon (Bruker, Version1.7.3, Kontich, Belgium), and analysis was performed using CTAn (Bruker, Version 1.13, Kontich, Belgium). The entire scaffold was selected as region of interest (ROI) for the analysis. Then, global thresholding was implemented for the binarization of images. Finally, using a built-in algorithm in CTAn for 3D structure analysis, the total porosity (Po, %) and connectivity density (Conn.D, 1/mm^3^) was calculated.

Diametral compression test were realized to evaluate the mechanical behavior of the scaffolds with pure HA and 50HA/50β-TCP with 20 wt.% and 40 wt.% of slurry solid content and sintered at 1150 °C and 1200 °C using a Universal Mechanical Testing Machine EMIC DL-3000 (Norwood, MA, USA). For the test, a load cell of 1000 N at room temperature was used. The dimensions of each sample (diameter/height ratio ≥ 2) were measured prior to the test. Assays were carried out at a crosshead speed of 0.4 mm/min. The diametral compressive strength was calculated from the maximum compressive load and the respective surface area of each specimen. Five samples were tested, and the average and standard deviation were calculated. ANOVA statistical analysis was used to determine significant differences.

### 4.5. Bioactivity Tests

The bioactivity of HA scaffolds was explored by immersion in simulated body fluid (SBF) according to the ISO 23317: 2007 standard [51]. For these experiments, HA scaffolds with solid content between 20 wt.% to 40 wt.% sintered at 1100 °C, 1150 °C and 1200 °C were used. The SBF was prepared according to Kokubo’s protocol [52]. For this, the following reagents were used as received: NaCl (FARMOS, Rio de Janeiro, Brazil); NaHCO_3_ (QUIMESP, Guarulhos, Brazil), KCl (NEON, São Paulo, Brazil); K_2_HPO_4_*3H_2_O and MgCl_2_*6H_2_O (SPECTRUM, New Brunswick, NJ, USA), HCl (1M) and NH_2_C(CH_2_OH)_3_ (MERCK, Haar, Germany); CaCl_2_ and Na_2_SO_4_ (DINAMICA, Indaiatuba, Brazil). All above-listed components were mixed in the appropriate order and quantities, and the final pH was adjusted to 7.25. The test was carried out by immersion of the scaffolds in the SBF solution and further incubation at 37 °C for 30 days. After immersion time, samples were gently washed three times with ddH_2_O and dried at 60 °C for 48 h. Changes in the surface morphology of the scaffolds was analyzed by field emission scanning electron microscopy (FESEM, Quanta FEG, 450, Hillsboro, OR, USA) with the same procedure of described in Section 4.4.

### 4.6. In Vitro Biocompatibility

The biocompatibility of the scaffolds fabricated from CaP nanoparticles was evaluated by culturing human adipose mesenchymal stem cells on the scaffolds. For this, human adipose mesenchymal stem cells were isolated from human adipose tissue obtained from healthy donors (*N* = 3). Tissue was harvested after obtaining an informed patient’s written consent. This study was approved by the Local Ethics Committee of the ‘‘Klinikum Rechts der Isar’’ at the Technical University of Munich, Germany.

Isolation of hAMSCs from fat tissue was performed as reported in [53]. Briefly, small pieces of fat tissue were centrifuged (430 g) to separate the stromal fraction. After centrifugation, fat tissue was digested with 1.45% collagenase solution (Merck Millipore, Haar, Germany) for 30 min at 37 °C and centrifuged at 600 g to obtain a cell pellet. Cells were cultured in DMEM medium supplemented with 10% fetal calf serum (FCS) and 1% penicillin/streptomycin (P/S) at 37 °C and 5% CO_2_ in a humidified atmosphere. Medium was changed twice a week and cells were passaged when reaching confluence. Unless otherwise indicated, all cell culture components were from SIGMA-ALDRICH (St. Louis, MO, USA).

Prior to cell seeding, the scaffolds were sterilized by three subsequent immersions in 70% ethanol. Thereafter, the scaffolds were allowed to air dry in the cell culture hood for a minimum of 4 h and thoroughly washed with sterile cell culture medium. For cell seeding on the scaffolds, 1 × 10^5^ cells were used. To improve cell attachment and eliminate any ethanol residue, scaffolds were incubated in cell culture medium overnight before seeding. Cells were seeded using a volume of 50 μL/scaffold. Cells were left to attach for 2 h at 37 °C before replenishing with fresh medium.

Cell viability was evaluated by lactate dehydrogenase (LDH) assay using a Fluitest LDH-L Kit (ANALYTICON, Lichtenfels, Germany) and following the instructions of the manufacturer. In brief, cell supernatants were harvested two and seven days after hAMSCs seeding on the scaffolds. Next, 75 µL of the cell supernatant was mixed with 100 µL of the kit working solution. This was performed in triplicates for each scaffold. Thereafter, absorbance was measured at 340 nm in a multiplate reader (FLUOstar Omega photometer (BMG labtech, Ortenberg, Germany).

In a similar experiment, cell proliferation was evaluated after 2, 7, and 14 days of cell culture on the scaffolds. For this, DNA was quantified by means of a PicoGreen assay using the Quant-iT PicoGreen dsDNA assay kit (Invitrogen, Carlsbad, CA, USA). The assay and further measurement was performed following the instructions manual. Briefly, equal volumes of cell lysate and assay kit working solution were pipetted into a 96-well plate. Next, the plate (light-protected) was incubated for 5 min at 37 °C. Thereafter, fluorescence was measured at 520 nm/485 nm in a multiplate reader. Experiments were performed in triplicate.

In order to visualize cell attachment and cell distribution on the scaffold, hAMSCs seeded scaffolds were harvested after 2, 7, and 14 days of culture. Next, scaffolds were carefully washed with sterile Phosphate-buffered saline (PBS) and fixed with 2.5% glutaraldehyde (AppliChem, Darmstadt, Germany) freshly prepared in cacodylate buffer (0.1M, pH 7.4). After a fixation period of 30 min, the samples were dehydrated by using solutions with increasing concentrations of ethanol. Incubation in hexamethyldisilazane was performed three times for 10 min each. Finally, the specimens were allowed to air-dry and sputter-coated with gold. SEM observation were performed in a Jeol JSM 5400 (Peabody, MA, USA).

## 5. Conclusions

Our results support that nanoparticles of HA and CDHA can be useful as starting materials for the fabrication of 3D sponge-like ceramic scaffolds. The resulting scaffolds featured high total porosity and pore interconnectivity. Moreover, through the control of sintering processing parameters, it was possible to prepare scaffolds with nanostructured topography, desired pore size and interconnectivity, and better mechanical properties. Interestingly, an increase in the HA solid content resulted in smaller pore size and negatively impacted the biocompatibility of the scaffolds. In this line, the composite 50HA/50β-TCP scaffolds featured good mechanical properties and highly improved biocompatibility. Overall, our results demonstrate how sintering processing parameters are important for directing mechanical properties and cell response. We presented here a highly porous ceramic scaffold with a high pore interconnection that was both bioactive and biocompatible, and may be favorable for bone tissue engineering applications.

## Figures and Tables

**Table 1:** Dependence of grain size, pores size, total porosity or density of interconnection of scaffolds obtained from CaP nanoparticles with different slurry solid content, sintering temperature or composition.

Sintering Temperature (°C)	Composition	Solid Content (wt.%)	Macroporous Size (μm)	Grain Size (μm)	Total Porosity (%)	Connectivity Density (1/mm^3^)
1200 ^a^		20	330 ± 180	1.6 ± 0.5	87.16	18.94
1200	HA	30	340 ± 160	1.7 ± 0.5	80.77	22.31
1200 ^b^		40	330 ± 150	1.6 ± 0.9	74.98	26.22
1100		20	550 ± 190	0.7 ± 0.3	-*	-*
1150	HA	20	445 ± 115	1.0 ± 0.4	79.80	22.30
1200 ^a^		20	330 ± 180	1.6 ± 0.5	87.16	18.94
1100	HA	40	430 ± 205	0.9 ± 0.2	71.53	24.61
1100	50HA/50 β-TCP	40	310 ± 125	bimodal	71.28	43.63
1200 ^b^	HA	40	330 ± 150	1.6 ± 0.9	74.98	26.22
1200	50HA/50 β-TCP	40	290 ± 100	bimodal	75.40	38.51
1200	β-TCP	40	280 ± 130	2.8 ± 1	-**	-**

^a, b^ Same samples; * Not tested: Sample with less solid content and less sintering temperature and therefore too brittle; ** Not tested: Sample with significant closed pores at surface.

**Table 2:** Compositions of scaffolds fabricated from CaP nanoparticles *.

**Part I**									
**Calcium phosphate particles**				**Solid Content in the slurry** **(wt.%)**			**Proportion** **HA/β-TCP** **(%)**	**Sintering** **Temperature** **(°C)**	
HA				20–40			100/0	800–1200	
CDHA **				20–40			0/100	800–1200	
HA/CDHA **				40			80/20 50/50 20/80	900–1200	
	
	
**Part II**									
**Samples**					**Tests**				
**Scaffolds** **HA/β-TCP**	**S.C** **(wt.%)**	**S.T** **(°C)**			**XRD**	**FESEM**	**MECHANICS**	**BIO-ACTIVITY**	**µCT/ LDH/ DNA**
100HA	20	1100,1150,1200			1200	✓	-	✓	1150,1200
	30	1100,1200			-	✓	-	✓	1200
	40	1100,1150,1200			1200	✓	1150,1200	✓	1100,1200
80HA/20β−ΤCP	20	1150, 1200			-	✓	✓	-	-
	40	1150, 1200			1200	✓	✓	-	-
50HA/50β−ΤCP	20	1150,1200			-	✓	✓	-	-
	40	1150,1200			1200	✓	✓	-	1100,1200
20HA/80β−ΤCP	2040	1150, 1200 1150, 1200			- 1200	✓ ✓	✓ ✓	- -	- -
100β-TCP	2040	1150, 1200 1150, 1200			1200 1200	✓ ✓	✓ ✓	- -	- -

* HA, hydroxyapatite; CDHA, calcium deficient hydroxyapatite; S.C, solid content; S.T., sintering temperature; XRD, X-Ray diffraction; FESEM, field emission scanning electron microscopy; µCT, micro computed tomography; LDH, lactate dehydrogenase; ** CDHA with temperature transforms into β-TCP; **✓** all compositions tested.

## Abbreviations

ACP	Amorphous calcium phosphate
BCP	Biphasic calcium phosphate
BET	Brunauer–Emmett–Teller
CaPs	Calcium phosphate
CDHA	Calcium deficient hydroxyapatite
DCS	Diametral compression strength
DLS	Dynamic Light Scattering
FESEM	Field Emission Scanning Microscopy
FTIR	Fourier Transformed Infrared spectroscopy
HA	Hydroxyapatite
hAMSCs	Human adipose mesenchymal stem cells
JCPDS	Joint Committee on Powder Diffraction Standards
LDH	Lactate Dehydrogenase
PAA	polyvinyl alcohol
PBS	Phosphate-buffered saline
SBF	Simulated Body Fluid
TEM	Transmission Electron Microscopy
XRD	X-Ray Diffraction
β-TCP	β-tricalcium phosphate
µCT	Micro Computed Tomography
